# Normalization of Gastrointestinal Symptoms in Adults With Constipation With Daily Green Kiwifruit Consumption: Protocol for an Open-Label Intervention Study

**DOI:** 10.2196/75286

**Published:** 2026-01-13

**Authors:** Jasjot Maggo, Hwei Min Ng, Simone Birgit Bayer, Catherine L Wall, Caroline L Hoad, Luca Marciani, Jane Mullaney, Diana Cabrera, Karl Fraser, Janine M Cooney, Catrin S Günther, Tania Trower, Jeffery Tang, Olivier Gasser, Amber Milan, Warren C McNabb, Robin Spiller, Tamlin Conner, Chris Frampton, Meika Foster, Nicole C Roy, Richard B Gearry

**Affiliations:** 1 Department of Medicine, Christchurch, University of Otago, 2 Riccarton Avenue, Christchurch, 8011, New Zealand, 64 3 244 1074; 2 High Value Nutrition National Science Challenge, Auckland, New Zealand; 3 NIHR (National Institute for Health and Care Research) Nottingham Digestive Diseases Biomedical Research Unit, Nottingham University Hospitals NHS (National Health Service) Trust and the University of Nottingham, Nottingham, United Kingdom; 4 Grasslands, AgResearch, Palmerston North, New Zealand; 5 Riddet Institute, Massey University, Palmerston North, New Zealand; 6 Plant and Food Research, Palmerston North, New Zealand; 7 Ruakura Research Centre, Plant and Food Research, Hamilton, New Zealand; 8 Malaghan Institute of Medical Research, Wellington, New Zealand; 9 Liggins Institute, University of Auckland, Auckland, New Zealand; 10 Department of Psychology, University of Otago, Christchurch, New Zealand; 11 Edible Research, Rangiora, New Zealand; 12 Department of Human Nutrition, University of Otago, Dunedin, New Zealand; 13 Te Whatu Ora Waitaha, Canterbury, Christchurch, New Zealand

**Keywords:** functional constipation, constipation-predominant irritable bowel syndrome, green kiwifruit, abdominal pain, colonic volume, gut microbiome, metabolites, randomized control trial, mobile phone

## Abstract

**Background:**

Irritable bowel syndrome with constipation (IBS-C) and functional constipation (FC) have significant personal, health care, and social impacts, affecting patients’ quality of life. Treatment of these conditions is challenging. While green kiwifruit is a promising natural alternative to laxatives, its effectiveness in managing abdominal pain and the underlying mechanism of action is yet to be substantiated.

**Objective:**

This study investigates the effect of consuming 2 green kiwifruit daily for 4 weeks (the habitual serving) on abdominal pain and discomfort in individuals with IBS-C and FC.

**Methods:**

This study is a 2-arm parallel, open-label, placebo-controlled randomized study. This study’s duration was 9 weeks, with a 3-week lead-in phase, a 4-week intervention phase, and a 2-week follow-up phase. A total of 60 participants with IBS-C and FC were randomized to consume either 2 Zespri green kiwifruit (*Actinidia deliciosa* “Hayward,” ~150 g per serving, ~90 kcal) or maltodextrin (calorie-matched to the fruit, ~25 g per serving, ~90 kcal) per day for 4 weeks. The participants completed validated questionnaires assessing digestive and general health and well-being parameters, underwent magnetic resonance imaging to determine colon physiological measures, ingested a blue food dye, provided blood and fecal samples to measure microbial, immunological, and biochemical parameters, and ingested wireless motility devices (selected participants only) to assess physiological processes.

**Results:**

Recruitment for this study began in May 2021 and was completed in May 2022. A total of 63 participants were randomized, and 57 were analyzed using intention-to-treat analysis. Data analysis is complete, and full results are expected to be published in a peer-reviewed journal by April 2026.

**Conclusions:**

This study aims to evaluate the effectiveness of green kiwifruit consumption in managing abdominal pain in individuals with IBS-C and FC. It will provide new insights into the mechanisms behind the habitual consumption of green kiwifruit for digestive comfort in this population.

## Introduction

### Background

Chronic constipation affects about 14% of the population and has a substantial socioeconomic burden [1]. The symptoms of constipation include infrequent bowel movements, excessive straining, hard feces, and unsatisfactory defecation experience, with or without abdominal pain [1,2].

The 2 main constipation subtypes are irritable bowel syndrome with constipation (IBS-C) and functional constipation (FC). The key difference between the 2 is the presence of abdominal pain for patients with IBS-C [3]. In addition to constipation symptoms, these conditions are present with abdominal discomfort, bloating, reflux, indigestion, nausea, and vomiting [4]. They are frequently associated with psychological comorbidity, including mood disorders and anxiety, impacting quality of life [2,5,6]. Additionally, many individuals report extra-intestinal symptoms such as somatic pain, fatigue, and fibromyalgia [7-10].

Maintaining healthy dietary habits is essential for managing constipation. International guidelines [11-13] recommend drinking plenty of fluids and incorporating high-fiber foods (eg, fruits, vegetables, whole-grain bread, and cereals) into the diet as a first-line treatment for constipation. Moreover, many individuals prefer to self-manage their constipation symptoms with dietary management as opposed to pharmacological drugs [14].

Green kiwifruit (*Actinidia deliciosa* “Hayward”) is known for its laxative effects [15]. Several interventional studies of fresh fruit have reported improvements in bowel movement frequency, consistency, and ease of defecation [16-22]. Three studies involving participants with constipation have reported a significant improvement in complete spontaneous bowel movements [17,20,21]. Two recent studies demonstrated improved abdominal pain in people with constipation [20,21]. Additionally, some short-term studies have shown that consuming 2 servings of green kiwifruit can improve sleep quality, lipid profile, blood pressure, and platelet aggregation [23-25].

Green kiwifruit is rich in dietary fiber (2%‐3%), which is primarily derived from plant cell walls. The fiber has unique hydration properties, such as water retention, swelling, and viscosity [15,26-28]. A recent magnetic resonance imaging (MRI) study by Wilkinson-Smith et al [29], 2019, found that eating 2 servings of green kiwifruit twice a day for 3 days increased the water content of both the small bowel and the chyme in the ascending colon and raised colonic volume. These results support earlier in vitro research, which suggested that the polysaccharides in kiwifruit cell walls have a significant capacity for swelling and water retention. This indicates that kiwifruit may exert both osmotic and bulking effects in the colon [28,30].

Additionally, in vitro and animal studies have shown that the proteolytic enzyme actinidin from green kiwifruit aids gastric and small intestinal protein digestion [31,32], gastric emptying [33,34], and stimulates gut motility [35]. Only 1 human study has been published on the impact of green kiwifruit on the gut microbiome [36]. The study found that healthy individuals who consumed freeze-dried green kiwifruit (equivalent to 2 kiwifruit servings) for 4 days experienced a favorable shift in the abundance of lactic acid bacteria, such as lactobacilli and bifidobacteria, in fecal matter [36]. Moreover, there was a trend towards a decreased abundance of *Clostridium* and *Bacteroides* genera [36].

There is promising evidence from human, animal, and in vitro studies regarding the laxative effect of green kiwifruit. However, data on the effects of sustained consumption of 2 green kiwifruit daily on abdominal pain and the possible mechanisms of action in individuals with constipation are limited. This underscores the need for a comprehensive study to evaluate both the effectiveness and the underlying mechanisms involved.

### Hypotheses and Objectives

This study was based on the hypothesis that consuming 2 green kiwifruits daily over 4 weeks would improve abdominal pain.

Secondary hypotheses were that constipation and other associated symptoms improved. Additionally, the symptom improvement is due to improved physiological functions such as changes in colonic volume and water content, gut motility, gas fermentation profile, and biological functions such as the gut microbiome composition and the host-microbial metabolite profile.

The primary aim of this study was to assess the impact of consuming 2 green kiwifruits daily over 4 weeks on abdominal pain in participants with either FC or IBS-C.

The secondary aims were to determine the impact of consuming green kiwifruit daily over four weeks on (1) constipation and its associated symptoms using validated questionnaires; (2) bowel habits using a daily bowel movement diary; (3) mental health and general well-being parameters using validated questionnaires; (4) dietary fiber intake using validated questionnaires; (5) colonic volume and water content of colonic chyme using a validated MRI protocol; (6) whole gut transit time using blue dye; (7) a relative abundance of individual taxa, predictive function (gene abundances), and diversity indices of the fecal gut microbiota using shotgun metagenomic sequencing, fecal and plasma metabolites using liquid chromatography-mass spectrometry (LCMS); and (8) regional and whole gut transit time and gas fermentation profiles using Smartpill (Medtronic Inc) and Atmo gas-sensing capsules (Atmo Biosciences; Atmo capsule) in a subset of participants.

## Methods

### Study Design

This study was a 4-week, 2-arm, parallel, open-label, negative-controlled, randomized design investigating the daily impact of 2 Zespri green kiwifruit compared to calorie-matched maltodextrin on abdominal pain in 60 adults with IBS-C or FC. Thirty of these participants were invited to participate in the wireless motility devices (WMDs) substudy.

**Figure 1.**
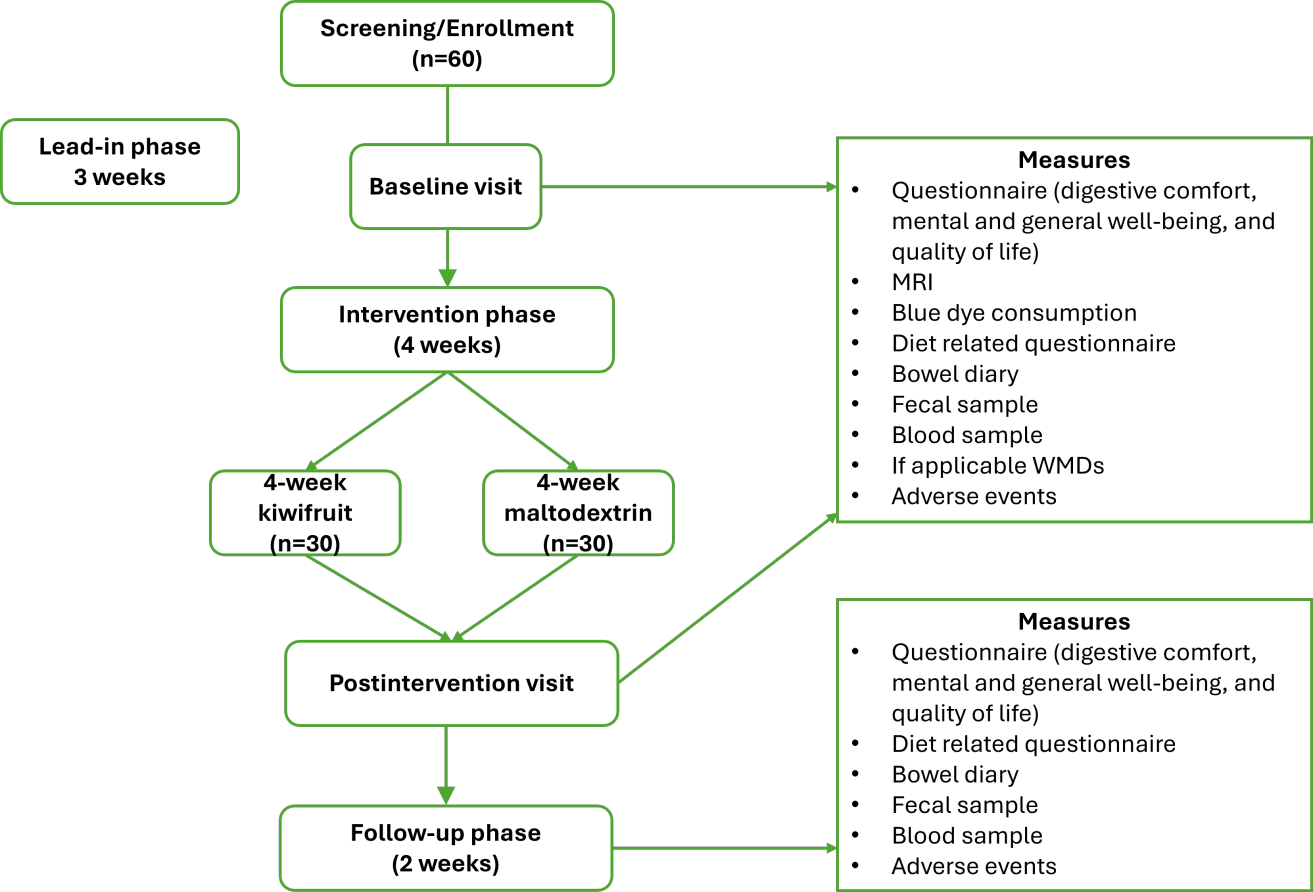
Study flowchart. MRI: magnetic resonance imaging; WMD: wireless motility device.

This study’s duration was 9 weeks: 21 days (3 weeks) lead-in phase, 28 days (4 weeks) intervention phase, and 14 days (2 weeks) follow-up phase (Figure 1). The lead-in phase started with participant enrollment and finished a week after the baseline visit. The week between the baseline visit and the intervention phase was designed to give sufficient time for the WMDs to be eliminated from the body [37]. The lead-in phase was followed by the intervention phase, where participants were either randomized to kiwifruit or maltodextrin. This was followed by a 2-week follow-up phase. This study was conducted according to the CONSORT (Consolidated Standards of Reporting Trials) and the SPIRIT (Standard Protocol Items: Recommendations for Interventional Trials) guidelines and adhered to the superiority study framework [38,39].

### Ethical Considerations

This study was approved by the Health and Disability Ethics Committee, New Zealand (NZ/21/NTB/96) May 14, 2021 and registered with the Australian New Zealand Clinical Trial Registry (ACTRN12621000621819) on May 24, 2021. The University of Otago Māori Health Advancement review process endorsed this study. Before entering this study, eligible participants provided informed consent, and additional informed consent was obtained for the WMDs substudy.

The researchers involved in data collection were experienced in maintaining the security and confidentiality of health information. This study did not report any identifying information about participants, such as names, birth dates, or images. Identifying details were collected during enrollment and accessed through REDCap (Research Electronic Data Capture; Vanderbilt University) only by the research team.

Participants received supermarket vouchers worth NZD $300 (US $185) as a thank you for completing the study. Additionally, the participants selected for the substudy received NZD $100 (US $62) to compensate for their time and any inconvenience.

### Recruitment

Participant recruitment started in May 2021 and ended in May 2022 in Christchurch, New Zealand. Due to slow recruitment at this study’s start, limited availability of MRI assessments, and a COVID-19 pandemic lockdown (August 2021-September 2021), study recruitment occurred over 2 kiwifruit growing seasons. Full results are expected to be published in peer-reviewed journals by the April 2026.

### Eligibility Criteria

#### Overview

All interested volunteers were assessed for eligibility based on the following criteria (Textbox 1).

Textbox 1. **Inclusion and exclusion criteria.**

Inclusion criteria

- Adults (18-65 years).
- BMI between 18-35 kg/m^2^.
- Function constipation or constipation-predominant irritable bowel syndrome as per ROME IV diagnostic criteria.

Exclusion criteria

- Significant gastrointestinal disorder.
- Alarm features associated with bowel habits.
- Known systematic disease that impacts the gastrointestinal tract directly or through medication.
- Significant chronic diseases.
- Allergy to kiwifruit or latex.
- Inability to access magnetic resonance imaging machine.
- Inability to swallow pills or stop laxatives.
- Irritable bowel syndrome severity score of over 300.
- Significant deviation for regular blood test.
- Blood glucose level of >6 mmol.

#### Inclusion Criteria

Adults (18‐65 y) with a BMI between 18 and 35 kg/m^2^, with either FC or IBS-C as per Rome IV diagnostic criteria, shown in Textbox 2, were recruited.

Textbox 2. **Rome IV diagnostic inclusion criteria for functional constipation (FC) and irritable bowel syndrome with constipation (IBS-C). Source: this textbox was generated using the study by Drossman [3].**

**FC (criteria fulfilled for the last 3 months with symptom onset at least 6 months before diagnosis)**

- Must include two or more of the following:
  - Straining during more than 25% of defecation.
  - Lumpy or hard feces (Bristol Stool Form Scale: 1 or 2) more than 25% of defecations.
  - Sensation of incomplete evacuation of more than 25% of defecations.
  - Sensation of anorectal obstruction or blockage of more than 25% of defecations.
  - Manual maneuvers to facilitate more than 25% of defecations.
  - Fewer than three spontaneous bowel movements per week.
  - Loose feces are rarely present without the use of laxatives.
  - Insufficient criteria for irritable bowel syndrome.

**Constipation-predominant irritable bowel syndrome (criteria fulfilled for the last 3 months, with symptom onset at least 6 months before diagnosis)**

- Recurrent abdominal pain on average at least 1 day/week in the last 3 months, associated with two or more of the following criteria:
  - Related to defecation.
  - Associated with a change in the frequency of feces.
  - Associated with a change in the form (appearance) of feces.
  - Criteria for constipation-predominant subtype.
  - More than ¼ (25%) of bowel movements with Bristol Stool Scale types 1 or 2.
  - Less than ¼ (25%) of bowel movements with Bristol Stool Scale feces types 6 or 7.

#### Exclusion Criteria

Participants were excluded if (1) they had a significant gastrointestinal disorder other than IBS-C (eg, diverticulitis, celiac disease, inflammatory bowel disease, or previous bowel resection); (2) alarm features associated with bowel habits such as a recent change in bowel habits (less than three months), rectal bleeding, sudden weight loss, fecal occult blood, anemia, anal fissures, bleeding hemorrhoids, and family history of early onset gastrointestinal cancer or inflammatory bowel disease; (3) any known systemic disease that impacts the gastrointestinal tract directly or through medication use; and (4) significant chronic diseases such as cardiovascular disease, cancer, renal failure, or neurological diseases. Additionally, people with a known allergy to kiwifruit or latex, inability to access an MRI machine, inability to swallow pills, inability or unwillingness to stop laxative use for 7 days (except for rescue medication bisacodyl suppositories), and a failure to comply with study procedures were also excluded. An irritable bowel syndrome severity score of over 300 and significant deviations in complete blood count, renal function, liver function, C-reactive protein, and fasting blood glucose of >6 mmol also resulted in exclusion.

#### Nonexclusion Criteria

Participants diagnosed with diabetes or conditions requiring selective serotonin reuptake inhibitors, tricyclic antidepressants, or nonsteroidal antiinflammatory drugs were recruited if the health condition and the medication use had been stable for over 3 months.

### Screening and Enrollment

Eligibility parameters were collected in 2 steps: participants completed a prescreening online questionnaire and an in-person screening visit. The prescreening questionnaire included questions based on inclusion and exclusion criteria, including questions related to the Rome IV diagnostic criteria (Textbox 2). Based on these replies, participants were diagnosed with either FC or IBS-C. Participants who met all study criteria were invited for a personal screening visit to provide consent and a blood sample to exclude any significant abnormalities.

Upon enrolling in this study, each participant was sent an individualized REDCap survey link hosted by the University of Otago, New Zealand, to complete 2 online enrollment questionnaires. One of the questionnaires was the Modified Hunter New England Health Survey [40], which includes the validated 12-item Short-Form Health Survey Version 2 (SF-12v2) and selected question domains from the New South Wales Population Health Survey.

The SF-12v2 questionnaire was used to evaluate the general health status of the participants, measuring eight domains of functioning and well-being, including physical functioning, role limitations due to physical problems, bodily pain, general health perception, energy and vitality, social functioning, role limitation due to emotional problems, and mental health well-being of the participants. The SF-12v2 questionnaire was scored by using published algorithms [41] to produce the physical component summary-12 and mental component summary-12. Participants also completed the Economic Living Standard Index short form. This validated questionnaire assesses the standard of living and socioeconomic background, ranging from “very good” to “severe hardship” [42].

### Study Intervention

Participants were randomly assigned to consume Zespri 2 green kiwifruit (*Actinidia deliciosa* cultivar “Hayward,” ~150 g per serving, ~90 kcal) per day or the calorie-matched negative control (Maltodextrin, 25 g per dose, ~90 kcal) for 4 weeks. The participants were instructed to consume 2 kiwifruit without skin or maltodextrin (available as powder) in 1 daily dose by incorporating it into their morning meal or breakfast. The intervention provided in this study was in addition to their usual diet.

Participants were provided with a minimum of a 14-day supply of kiwifruit on the day of the baseline visit and had their supply replenished for the subsequent 14 days. A few additional days’ supply was provided to the participants to ensure good-quality fruit was available, given that fresh fruit is a perishable product. Providing a 14-day supply was considered reasonable, as the shelf life of kiwifruit is typically around 16‐20 days at room temperature.

The kiwifruit was supplied by Zespri International Limited for the duration of this study.

The negative control was commercially sourced digestible maltodextrin (Davis Food Ingredients). This low-sweet polymer of D-dextrin was selected as a placebo as it can easily match the calorie content of the kiwifruit. Maltodextrin is also completely digested into glucose and actively absorbed in the small intestine [43], which does not increase small- or large-intestinal water content [29]. Participants receiving maltodextrin were asked not to consume kiwifruit during this study’s period.

### Randomization and Blinding

Following enrollment, participants were randomly allocated to the kiwifruit or maltodextrin group using randomized permuted blocks (block size four) drawn from sealed opaque envelopes. Due to the nature of the intervention, that is, consumption of whole fruit, blinding the participant and the administering researcher was not possible. The researchers involved in the analysis were blinded to the group allocation.

### Data and Sample Collection

**Figure 2.**
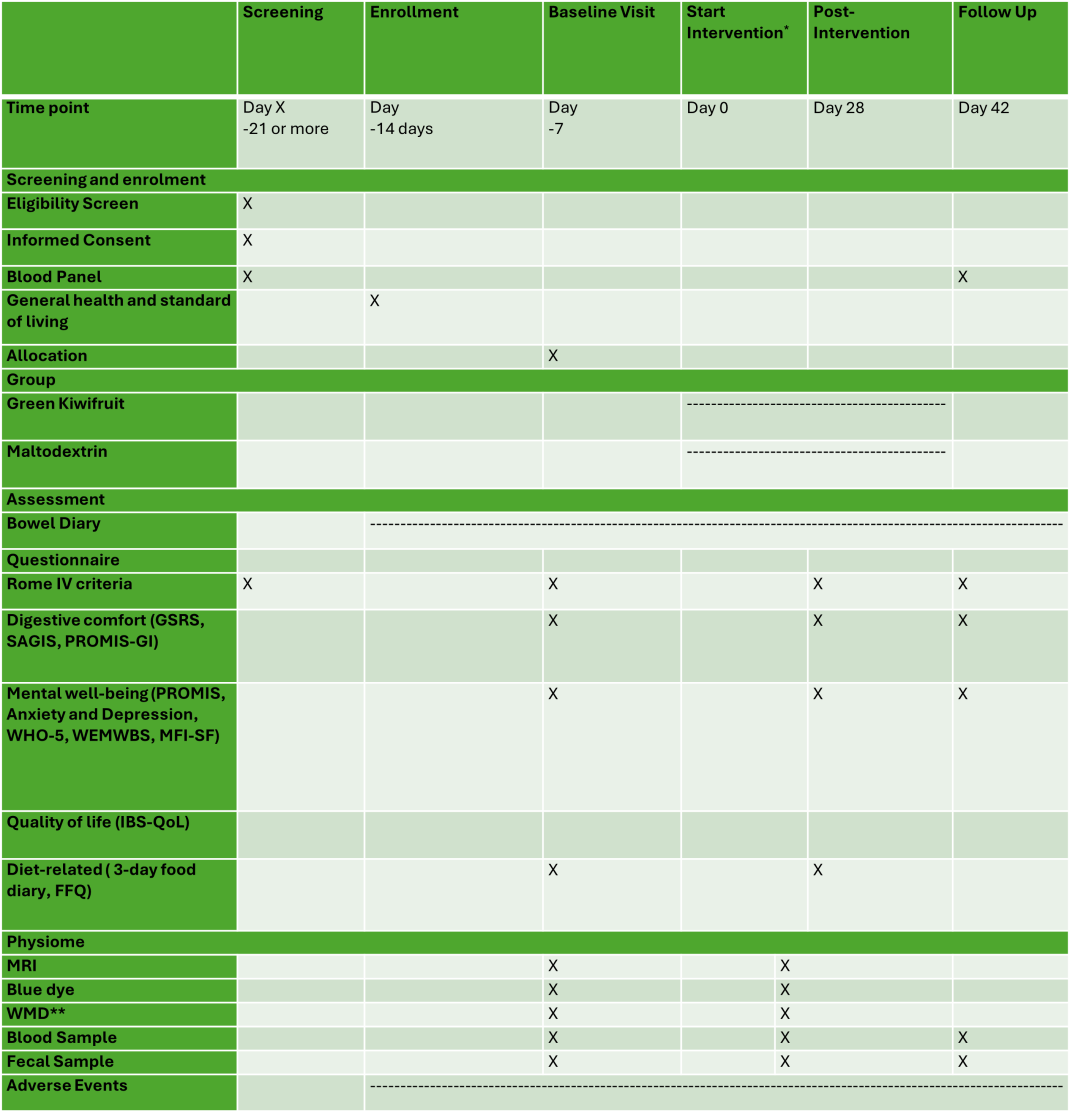
Overview of the schedule of screening, enrollment, interventions, and assessments. FFQ: Food Frequency Questionnaire; GSRS: Gastrointestinal Symptom Rating Scale; IBS-QoL: Irritable Bowel Syndrome Quality of Life Survey; MFI-SF: Multidimensional Fatigue Inventory Short Form; MRI: magnetic resonance imaging; PROMIS: Patient-Reported Outcomes Measurement Information System; PROMIS-GI: Gastrointestinal Domain of the Patient-Reported Outcomes Measurement Information System; SAGIS: Structured Assessment of Gastrointestinal Symptoms; WEMWBS: Warwick-Edinburgh Mental Well-Being Scales; WHO-5: World Health Organization - Five Question Well-Being Index; WMD: wireless motility device. *Participants began intervention 7 days after the baseline visit to ensure the excretion of the WMDwireless motility device.**A subset of participants completed this assessment.

Figure 2summarizes the timing of measures. Web-based questionnaires were administered to participants at different stages of this study, including screening, enrollment, baseline, postintervention, and follow-up assessments. Individualized REDCap survey links were sent to participants to complete questionnaires. A paper copy of the questionnaires was available on request.

Participants underwent an MRI assessment, ingested blue food dye, and provided blood and fecal samples at baseline and postintervention. In addition, participants completed a three-day food diary at baseline and postintervention. They also completed a follow-up visit 2 weeks postintervention, providing blood and fecal samples. Participants recorded daily bowel movement diaries using a REDCap-based app from enrollment until the follow-up period. A subset of participants also ingested WMDs at baseline and postintervention.

This study’s participants were given a cooler bag that contained a fecal collection kit and an ice pack. They were instructed to collect 2 fecal samples in the provided 60 mL containers (2 containers were provided) ≤24 hours before clinic visits at baseline, postintervention, and follow-up. One sample was stored at 4 °C in their home refrigerator and the other in their freezer (−18 °C) and transported within 24 hours in the provided cooler bag.

Extra fecal collection kits were offered to participants with less frequent bowel movements (occurring once in 3 days or less). They were encouraged to collect fecal samples 48 to 72 hours before and up to 24 hours after the clinic visit. Participants were also instructed to label the container with the collected time and date and record it in the bowel movement diary app. The collected samples were kept chilled until aliquoted in the laboratory. The home-frozen fecal sample was stored at −80 °C until the meta-transcriptomic analysis. The chilled fecal sample was aliquoted (1 g each) for analyses of microbial composition, microbial gene abundances, metabolome, bile acid, organic acid, and immune marker analyses. All aliquots were snap-frozen in liquid nitrogen before storage at −80 °C until analysis.

The participants also provided a blood sample (described below) at the in-person screening visit, baseline, postintervention, and follow-up. At the screening visit, blood samples were collected for complete blood count in a 4 mL ethylenediaminetetraacetic acid (EDTA) vacutainer and an 8 mL heparin tube for standard biochemistry analyses (liver function, renal function, electrolytes, C-reactive protein, and blood glucose) at Canterbury Health Laboratory (Christchurch, New Zealand). These analyses were repeated at follow-up visits before participants were discharged from this study.

At baseline and postintervention, blood samples were collected in one 4 mL EDTA tube, three 9 mL EDTA tubes, and one 6 mL heparin tube. The 4 mL EDTA and 6 mL heparin tubes were centrifuged for 5 minutes at 2000 x g with full acceleration and no deceleration. The plasma was aliquoted (4×500 µL) into labeled Eppendorf tubes and stored at −80 °C until metabolome, targeted metabolite, and immune profile analyses were performed. Peripheral blood mononuclear cells (PBMCs) were isolated from approximately 30 mL of whole blood (3×9 mL EDTA vacutainers) using the SepMate technique [44]. Briefly, the blood was split into two 15 mL centrifuge tubes and diluted in a 1:2 v/v ratio by adding 15 mL of Dulbecco’s phosphate-buffered saline containing 2% heat-inactivated fetal bovine serum to each tube. Next, the diluted blood was transferred into two SepMate tubes containing 15 mL of Lymphoprep. The SepMate tubes were centrifuged at 1200 x g for 10 minutes. After centrifugation, the top layer was poured into a new tube. This tube was centrifuged at 700 x g for 8 minutes, and the supernatant was removed. The pelleted PBMC was washed with 10 mL of Dulbecco’s phosphate-buffered saline containing 2% heat-inactivated fetal bovine serum and centrifuged again at 300 x g for 8 minutes, aspirating the supernatant while leaving the pelleted PBMC intact. Finally, the PBMC pellet was resuspended in 3 mL of a freezing mixture of 90% heat-inactivated fetal bovine serum and 10% (v/v) dimethyl sulfoxide and aliquoted into three cryovials. The cryovials were placed into a CoolCell LX freezer container and stored at −80 °C overnight. The following day, all the frozen cryovials were transferred to liquid nitrogen storage at −80 °C.

### Primary Outcome

The self-administered Gastrointestinal Symptom Rating Scale (GSRS, AstraZeneca) [45], a 15-item questionnaire, assessed common gastrointestinal symptoms, including abdominal pain, constipation, diarrhea, reflux, and indigestion. The abdominal pain domain of GSRS was used to determine the primary outcome of abdominal pain and discomfort relief. The GSRS is a 7-point Likert-type scale, where 1 represents the absence of symptoms, and 7 represents severe discomfort. The scale can be used as a whole, or the mean value for items under each symptom domain can be assessed to give a subscale [45]. The mean change in symptom scores between baseline and postintervention measurements was compared to evaluate the effectiveness of the intervention.

### Secondary Outcome

#### Physiological Parameters

##### About MRI

The MRI images were acquired using a MAGNETOM Skyra 3T scanner (Siemens Healthcare) by Pacific Radiology, and MRI image analysis was completed by members of the Nottingham Biomedical Research Center using a noninvasive MRI technique described elsewhere [29]. Briefly, participants were instructed to eat a standardized dinner the day before and fast overnight. They were then positioned supine in the scanner, and the colon was imaged using a variety of breath-holding scanning sequences.

The colonic volumes were assessed using a coronal 3D DIXON dual-echo sequence with a field of view (400×400 mm^2^) positioned to visualize the large intestine, from the splenic flexure down to all the colonic regions in the pelvis. Slices (48 in number) of 3.5 mm thickness covered the abdomen from anterior to posterior. The in-plane reconstructed resolution was 0.78×0.78 mm^2^ with a matrix of 512 ×512. Four image types were derived from the data, which provided images of water only, fat only, fat and water in phase, and fat and water out of phase. Individual regional colon volumes were manually segmented on each slice using Medical Image Processing, Analysis, and Visualization software (National Institute of Health) [46].

The colon of the participants was divided into 4 segments: ascending colon, transverse colon, descending colon, sigmoid colon, and rectum region. Each colon segment was identified within each coronal image slice, and a region of interest (ROI) was drawn around it, building a 3D representation of the morphology. The volume of each segment was measured by summing the areas of the corresponding ROI and multiplying by slice thickness. The total colon volume was calculated by summing the volumes of the 4 separate segments.

The relaxation time (T1), a time constant determining the rate at which magnetically excited water hydrogen protons in the colonic chyme take to return to equilibrium, was measured for the ascending and descending colon and reflects the water content of the chyme [47]. Two sagittal-oblique True Fast Imaging With Steady-State Free Precession (TRUFI) sequences were used to localize the ascending and descending colon, respectively.

Each colonic segment was scanned using an inversion recovery single-slice sagittal oblique TRUFI sequence, using 8 different inversion times ranging from 100‐5000 ms. Each different inversion time required a separate breath-hold, with at least a 15-second gap between each to allow full signal recovery before the subsequent acquisition. The field of vision was 284×400 mm^2^ with a slice thickness of 7 mm, with a reconstructed in-plane resolution of 1.56×1.56 mm^2^ and a matrix size of 182×256.

ROIs were drawn on all images in the chyme at 3 locations in each segment using custom-written software in Matlab (The MathWorks Inc). The mean signal in each ROI was measured at each inversion time. The data for each ROI was fitted to a model of the expected signal intensities in the inversion recovery–TRUFI sequence to determine the T1 as previously described [47]. Single values of T1 for the ascending and descending colon, respectively, were calculated from the average T1 of the 3 regions in that segment.

##### Blue Food Dye

Participants ingested Royal Blue Liqua-gel (Chefmaster) food coloring (12 drops/1.5 g) in water to measure the total gut transit time [48]. The time of ingestion was recorded, and participants were requested to log the first appearance of “blue/green feces” on the app-based bowel diary. The date and time (h, min) of the blue color’s appearance were recorded through this app. The time of ingestion and exit was used to calculate the whole gut transit time.

### Patient Reported Outcome

#### Constipation and Associated Symptoms

Constipation and its associated symptoms were assessed by validated questionnaires, including GSRS, the Rome IV questionnaire [3] Structured Assessment of Gastrointestinal Symptoms (Universities of Queensland and Newcastle) [49] and the Gastrointestinal Domain of the Patient-Reported Outcomes Measurement Information System (Health Measures) [50]. The Rome IV questionnaire classifies participants into IBS-C and FC groups and evaluates for the shift into a different classification during this study’s period. Structured Assessment of Gastrointestinal Symptom measures the intensity and impact of 22 upper and lower gastrointestinal symptoms and the presence of selected nongastrointestinal symptoms. Four study-relevant domains (disrupted swallowing, constipation, belly pain, and gas, bloating, or flatulence) of the Gastrointestinal Domain of the Patient-Reported Outcomes Measurement Information System were administered to assess gastrointestinal symptoms and group effects.

#### Bowel Movement Diary, Constipation, and Associated Symptoms

A REDCap app-based daily bowel habit diary captured data regarding bowel movement frequency, spontaneity, completeness, ease of defecation, use of manual maneuvers, Bristol Stool Scale, and menstruation (if applicable). The participants were asked to report the following after each bowel movement. This provided a comprehensive record of bowel habits and was used to determine the number of complete spontaneous bowel movements per week, defined as a complete evacuation not induced by laxatives, rescue medications, manual maneuvers, or enemas [21]. Other parameters include total bowel movement per week, average Bristol Stool Scale, and straining. In addition, this app was used to collect the time of excretion of blue food dye.

#### Mental Health and General Well-Being

General and mental well-being parameters were assessed by the World Health Organization - Five Question Well-Being Index (WHO-5) [51], the Warwick-Edinburgh Mental Well-Being Scale [52], the Multidimensional Fatigue Inventory Short Form [53], and Patient-Reported Outcomes Measurement Information System (PROMIS) anxiety and depression domain questionnaires [54].

The 5-item WHO-5 assesses recent experiences of well-being related to physical vitality, cheerfulness, and interest. The 14-item Warwick-Edinburgh Mental Well-Being Scale assesses psychological functioning (optimism, autonomy, agency, curiosity, clarity of thought, and positive relationships) and positive affect (confidence, feeling relaxed, cheerful, and having the energy to spare). The 30-item Multidimensional Fatigue Inventory Short Form measures various dimensions of fatigue (general fatigue, physical fatigue, emotional fatigue, mental fatigue, and vigor). The 8-item PROMIS depression questionnaire Version 8a assesses common symptoms of depression, such as worthlessness, depressed mood, and anhedonia. The 8-item PROMIS anxiety questionnaire Version 8a assesses common symptoms of anxiety, such as nervous mood, worries, and tension. In addition, the 34-item Irritable Bowel Syndrome - Quality of Life Survey measured the impact of irritable bowel syndrome and its management [55].

All questionnaires queried symptoms over the past week following their standard timeframes. The WHO-5 timeframe was modified from the past 2 weeks to the past week to maintain consistency with the other questionnaires.

#### Dietary Intake

All enrolled participants completed a nonconsecutive 3-day food diary before each study visit, that is, at baseline, postintervention, and follow-up. The food diary included written prompts for detailed information about when, where, and with whom food was consumed, the type of food or drink, brand details, preparation or cooking methods, quantity, and a section for recipe details. Participants were also asked to record if their intake differed from their normal eating routines. At each clinic visit, this study’s dietitian reviewed each food diary. Two nutrition graduates trained in dietary assessment entered the food diaries into a dietary analysis software, FoodWorks Online Professional (Version 1.0, Xyris Software, 2021). The NZ Food Composition Data (FOODfiles 2018 – Version 1.0) was used to estimate energy and nutrient intake.

In addition to the food diaries, participants completed a fiber-specific Food Frequency Questionnaire (FFQ) before each clinic visit. This FFQ assessed inulin and oligofructose consumption from 9 commonly eaten foods: wheat, onion, garlic, banana, leek, rye, asparagus, chicory, and barley [56]. The FFQ data were entered into a spreadsheet. Once all the data were collected, the spreadsheet was processed by a bespoke algorithm to calculate inulin and oligofructose consumption and the relative contributions of each food item.

### Biological Measurements

#### Fecal Microbiome

The taxonomic composition and gene abundances of the fecal microbiome were analyzed by shotgun metagenomics. Auckland Genomics constructed libraries for metagenomics sequencing of the extracted DNA at the University of Auckland, New Zealand. Microbial metagenomic libraries were then generated using the Seqwell PurePlex DNA library preparation approach [57]. Briefly, DNA was extracted from fecal samples using ZYMOBiomics DNA and ribonucleic acid Miniprep kits following the manufacturer’s instructions, with the addition of bead beating on a BioSpec Mini-Beadbeater 96 set to 4 minutes. DNA was measured using the Qubit high-sensitivity kit. DNA was normalized to 5 ng/µl using the Eppendorf Epmotion robot. The DNA was prepared for Illumina shotgun sequencing using the Seqwell Pureplex Unique Dual Index library preparation kit (Part No. 301067 for Set 1A; 301068 for Set 2B). The samples were shotgun sequenced using the Illumina Novaseq 6000 platform using 2 ×150 bp paired-end sequencing at Novogene in Singapore.

The raw data quality was checked using FastQC (V0.11.9). The software Trimmomatic (V0.36) [58] was then used for the removal of adapters, low-quality (Phred scores <30), and short (<36 bp) sequencing reads. Read pairs were aligned to the human reference genome (RefSeq: GCF_000001405) [59] using the “mem” algorithm of Burrow-Wheeler Aligner (V0.7.17-r1188) [60]. The unmapped sequencing reads were converted into fastq files using the “fastq” function of samtools (V1.8) [61]. Read pairs were joined using paired-end read merger (V0.9.6) [62] with default settings. Read pairs that did not join were pasted together with a string of N’s using the “fuse” function from the *BBMAP* package (V38.22‐0) [63]. Joined and fused reads from different lanes from the same sample were compiled into a final “clean” read sample file. Metagenomic functions were obtained through the “blastx” function of Diamond (version 0.9.22) [64], mapping the reads against the “non-redundant” National Center for Biotechnology Information database [65]. Megan (version 6 ultimate edition) [66] was used to assign putative functions to the alignment files produced by Diamond. A trained statistician (AgResearch) performed this alignment.

Differential abundance was performed using DESeq2 in R software (version 1.40.2; R Foundation for Statistical Computing). This package analyzes differentially expressed taxa based on a negative binomial distribution. The effect of groups was assessed by comparing between time points and groups. The top 10 differentially abundant taxa were extracted based on false discovery rate (FDR) adjusted *P* values for both baseline and postintervention for each group. Taxa that changed between the two time points within each group were identified by finding the set difference between the top taxa between baseline and groups postintervention. Log2 fold change values were extracted for the identified changed taxa in each group. Enrichment of functional gene attributes arranged hierarchically with level 1 (broadest level of function, eg, metabolism and cellular processes), level 2 (specific functions, eg, carbohydrate metabolism and amino acid metabolism), and level 3 (detailed pathways, eg, glycolysis and glycan degradation), was analyzed.

#### Plasma and Fecal Metabolome

Fecal samples were freeze-dried and extracted using a previously described method [67] with minor modifications. Briefly, 50 mg of lyophilized fecal samples were homogenized with a ceramic bead for 1 minute, and then 400 µL of 75% methanol/MilliQ water was added. The mixture was vortexed (30 s), sonicated (2 min), and transferred to ice for 10 minutes. Next, 1 mL of methyl tert-butyl ether was added, and the resulting mixture was incubated for 1 hour at 450 rpm. Then, 550 µL of MilliQ water was added, and the mixture was incubated (10 min) before being centrifuged at 14,000 x g for 25 minutes to separate the aqueous (lower) and organic (upper) phases. The polar and lipid extracts were evaporated under a stream of nitrogen at room temperature, and the resulting dried samples were stored at –80 °C until subjected to LCMS analysis. The metabolite profiling analyses were conducted using high-resolution LCMS on a Shimadzu Q-Tof 9030 with electrospray [68]. The polar extract was analyzed using hydrophilic interaction LC [68], and semipolar metabolites were resolved using reverse-phase liquid chromatography [69]. The lipidomic methodology was used to analyze the phase extract [70].

Polar, semipolar, and nonpolar metabolites were extracted from plasma using a previously described method [69]. Briefly, polar metabolites were extracted from 50 µL of plasma with 450 µL of prechilled acetonitrile: water (9:1 v/v). The mixture was shaken for 60 seconds before being centrifuged at 14,000 g at 4 °C for 10 minutes, and then the extract was placed into a high-pressure liquid chromatography (HPLC) vial for analysis. For semipolar metabolite extraction, 400 µL of ice-cold chloroform:methanol (1:1 v/v) was added to 50 µL of plasma, the mixture was then vortexed (30 s) and incubated (1 h) at −20 °C [71]. Then, 200 µL of MilliQ water was added to the mixture, vortexed, and centrifuged at 14,000 g (10 min) at 4 °C. The supernatants were evaporated to dryness under a stream of nitrogen and stored at −80 °C until analysis.

On the analysis day, the semipolar extracts were thawed at room temperature (18±2 °C) and redissolved in acetonitrile:water (1:9 v/v). The mixture was vortexed (1 min) and centrifuged (14,000 g, 4 °C, 10 min). The extract was transferred into an HPLC vial for analysis. For lipid extraction, 10 µL of plasma was mixed with 95 µL of butanol:methanol (1:1 v/v) and spiked with 5 µL of internal standard SPLASH mix (Avanti Lipids) [70]. The mixture was then vortexed (1 min), sonicated (60 min) at room temperature, and centrifuged at 14,000 g (10 min) at 20 °C. All extracts were transferred into an HPLC vial and stored at −80 °C until LCMS analysis. The plasma polar, semipolar, and nonpolar extracts were analyzed using the LCMS methods described for the fecal samples [70].

All LCMS data files were obtained in Data Independent Acquisition mode, facilitating mass spectrometry (MS)/MS spectral annotation and identification. The resulting data files were processed using MS-DIAL and publicly available databases [72]. MS-DIAL performs peak detection, retention time alignment, grouping, gap filling, run order, and batch correction using the LOWESS algorithm and then searches and annotates the aligned peaks found in the collected Data Independent Acquisition MS/MS spectral data.

The lipidome results were matched against the built-in lipid library containing 257,000 silico*-*generated MS/MS lipid fragmentation spectra. Semipolar and polar compounds were searched against the MS/MS public library, which included 13,303 unique compounds. This data matrix was subsequently exported to filter unreliably measured peaks using the quality control samples (relative SD>0.3). Furthermore, any peaks that occurred in the samples and blanks that were less than 3 times the size of the average peak in the blank were removed. The resultant data matrix was ready for downstream statistical analyses.

#### Plasma and Fecal Bile Acid

Extraction methods for bile acid analysis followed those previously outlined. Briefly, 50 mg of wet fecal samples were extracted with 100 µL ice-cold methanol containing internal standards (10,000 nM of d_5_-CA and d_5_-CDCA). After homogenizing the mixture for 30 s, it was incubated at −4 °C for 30 minutes and then centrifuged at 18,000 g for 20 minutes. The supernatant (20 µL) was then dissolved in 80 µL of 0.1% aqueous formic acid solution and vortexed before being analyzed using LCMS on an AB Sciex LCMS/MS QTRAP 6500+ system (Sciex) coupled to an ExionLC (Shimadzu) [73]. Bile acid peak areas were measured, and absolute concentrations (ng/g dry fecal matter) were calculated for each bile acid based on the instrument response of known concentrations of each bile acid relative to the deuterated bile acid internal standards.

#### Plasma and Fecal Organic Acids

Aliquots of 200 μL of heparin plasma and 1 g of fecal samples were sent to Plant & Food Research to measure organic acid concentrations using an LCMS method. Fourteen linear and branched organic acids (carbons 1 to 6) were derivatized with an MS probe and stable isotope techniques as previously described [74] with modifications [75,76]. The names and corresponding acronyms of organic acids are presented in Table 1. Plasma organic acids were measured using targeted LCMS on an AB Sciex LCMS/MS QTRAP 7500 instrument equipped with a Turbo V ion source and atmospheric pressure chemical ionization probe (Sciex) coupled to a Nexera UHPLC (Shimadzu). Fecal organic acids were measured using targeted LCMS on an AB Sciex LCMS/MS QTRAP 5500 instrument equipped with a Turbo V ion source and atmospheric pressure chemical ionization probe (Sciex) coupled to an Exion UHPLC (Shimadzu). Labeled internal standards for each organic acid were used to ensure accurate quantification.

**Table 1.** Plasma and fecal organic acids were analyzed using LCMS with the corresponding acronym.

Full name	Acronym
Formic acid	FA
Lactic acid	LA
Acetic acid	AA
Propanoic acid	PA
Isobutyric acid (2-methylpropanoic acid)	IBA
Butyric acid (butanoic acid)	BA
Succinic acid	SucA
2-methyl butyric acid (2-methylbutanoic acid)	2MBA
Isovaleric acid (3-methylbutanoic acid)	IVA
Valeric acid (pentanoic acid)	VA
3-methyl valeric acid	3MVA
4-methyl valeric acid (isocaproic acid)	4MVA
Caproic acid	CA
Hexanoic acid	HA

a LCMS: liquid chromatography-mass spectrometry.

#### Plasma Neurotransmitters

Two aliquots of 500 μL of heparin plasma were sent to Plant and Food Research for quantitative measurement of neurotransmitters from the tyrosine and tryptophan metabolic pathways identified as important to the Gut-Brain Axis using two separate LCMS methods as previously described [77]. Method A uses an MS-probe and stable isotope coding derivatization LCMS method to quantitatively convert neurotransmitters and related compounds into their corresponding acetate or ester, thereby increasing their analytical sensitivity. Method B is used to quantify kynurenic acid, quinolinic acid, and xanthurenic acid, which are poorly derivatized using method A. These three analytes and their labeled internal standards are measured directly by LCMS and are underivatized. The names and corresponding acronyms of neurotransmitters are presented in Table 2. Plasma neurotransmitters were measured using targeted LCMS on an AB Sciex LCMS/MS QTRAP 7500 instrument equipped with a Turbo V ion source and APCI probe (Sciex) coupled to a Nexera UHPLC (Shimadzu).

**Table 2.** Plasma neurotransmitters analyzed using LCMS^a^ with the corresponding acronym.

Full name	Acronym
Serotonin	5HT
L-kynurenine	KYN
Tryptophan	TRP
Quinolinic acid	QA
DL- 5 hydroxytryptophan	5HTP-DL
Xanthurenic acid	XA
Kyruneric acid	KA
Hydroxy-indoleacetic acid	5-HIAA

a LCMS: liquid chromatography-mass spectrometry.

#### Plasma Immune Markers

Two EDTA plasma and 3 aliquots of isolated PBMC were sent to the Malaghan Institute of Medical Research for plasma cytokine and immune profile analysis. The concentration of circulating cytokines, including IL-1β, IL-6, IL-8, IL-10, IL-12p70, IL-17A, IL-18, IL-23, IL-33, interferon-α2, interferon-γ, tumor necrosis factor-α, and monocyte chemoattractant protein-1 was measured using Human Inflammation Panel 1 LegendPlex assay (Cat #: 740809, BioLegend) as per manufacturer instructions. The PBMCs were counted using flow cytometry with a commercial staining kit to capture their activation state.

### About WMDs

During the main study, participants were invited to ingest a Smartpill and an Atmo capsule. The dual WMD ingestion protocol was similar to a previously described protocol [78]. Participants consumed a standardized cereal bar (Smart bar, Medtronic Inc) followed by the WMD with water. They fasted for 6 hours postingestion and wore transponders across their body (or close to their body at night) until WMD excretion. The participants were asked to enter additional bowel movement data on the Smartpill and Atmo capsule transponders for the duration of the substudy. Participants with gut transit times exceeding the capsule’s or transponder’s battery life (~5 d) were requested to visualize their feces to confirm the excretion of the capsule. If the excretion of one or both capsules could not be determined from the transponder data or visual confirmation, an abdominal radiological examination was conducted.

Smartpill measures anatomical landmarks and regional transit time via intraluminal temperature, pH, and pressure changes as it passes through the gastrointestinal tract [79]. The novel Atmo capsule measures anatomical landmarks and colonic fermentation by measuring oxygen, hydrogen, carbon dioxide, volatile organic compounds, capsule orientation, environmental electromagnetic properties, and temperature changes [78]. The analyses included regional transit time and whole-gut transit time measured by SmartPill and Atmo capsule, and colonic gas (hydrogen and carbon dioxide) profiles.

Smartpill transponder data were downloaded and analyzed using the proprietary software MotiliGI (version 2.5, Medtronic Inc). Atmo capsule transponder data was collected in real time on the smartphone app and transmitted to the Atmo cloud (hosted by Atmo Bioscience) for review and analysis.

### Adverse Events

All participants were informed about potential side effects. They were encouraged to record any symptoms and contact their health care provider if needed.

### Compliance

In this study, compliance was not formally measured. Participants received small batches of the intervention throughout this study’s period and were encouraged to continue consuming the interventional product. The food diaries collected during the postintervention visit provided insights into the consumption of kiwifruit and maltodextrin.

### Sample Size

This study was powered to detect an effect size of 1 within the abdominal pain domain of the GSRS, 5% significance (α), and 80% power (β)=16 participants regarding a difference between the groups based on GSRS data obtained from studies with gold kiwifruit [80-82]. In addition to the primary outcome, this study was intentionally powered to ensure the detection of differences between groups in colonic volume as measured by MRI as a secondary outcome. Based on a previous study with 300 g of kiwifruit, a parallel design with 5% significance (α) and 90% power (β), the minimum sample size was determined as 22 participants per group [29]. To account for the lower daily kiwifruit intake (150 g) over 4 weeks and an estimated dropout rate of 10%, a total of 30 participants needed to be recruited for each group.

### Statistical Analysis

This study was conducted as a superiority trial. The Guidelines of the Committee for Proprietary Medicinal Products (now termed Committee for Medicinal Products for Human Use) require intention to treat analysis [83]. All data from randomized participants who received at least one intervention dose and had at least one postintervention measurement were analyzed. All statistical analyses were performed using SPSS software (version 29.0; IBM Corp) or R software (version 4.3.1; R Core Team 2021) by blinded researchers under the guidance of an independent biostatistician.

Baseline characteristics were presented using descriptive statistics. The mean and SD, or median and range, were used to describe continuous variables. Frequencies and percentages were used to describe categorical variables. The group effects (ie, the mean change from baseline between groups) were assessed using (parametric) *t* tests and (nonparametric) Mann-Whitney or Kruskal-Wallis tests for symmetrically and asymmetrically distributed data. The mean, SD, and CIs were used to describe continuous variables. Categorical variables were assessed using chi-square tests (or Fisher exact tests for small samples). A 2-tailed statistical significance *P*<.05 showed statistical significance. A *P* value of <.1 would be considered a trend for most variables.

To analyze the fecal microbiome measures, both univariate and multivariate statistical methods were used to evaluate variations in alpha and beta diversity and differences in the relative abundance of taxa and gene abundance differences between groups. Differences in alpha diversity were assessed using the nonparametric Wilcoxon signed rank test with continuity correction, comparing baseline and postintervention data, as well as between groups postintervention. The Benjamini-Hochberg method was applied to control for the FDR associated with multiple hypothesis testing for variables in microbiome analyses. Adjusted *P* values were calculated to mitigate the inflated type I error rates arising from numerous comparisons. A *P*<.05 and FDR of *P*<.1 were considered significant, with a probable biologically significant difference (trend) at unadjusted *P*≤.1 indicating a trend.

Boxplots were created using ggplot2 to compare groups for each α diversity (Chao1 and Shannon indices) across time points. The statistical significance of microbial community composition was assessed using the permutational multivariate ANOVA and the Analysis of Dissimilarity (number of permutations: 999), which uses permutation testing and F tests to evaluate differences in beta diversity. Differential in microbial gene expression was analyzed using quasi-likelihood F tests, controlling for dispersion uncertainty. Genes with an absolute log-fold change greater than 1.1 were deemed differentially expressed.

As mentioned above, for plasma or fecal metabolome measures, the comparisons were between each group and baseline and between groups postintervention. The partial least squares projection-discriminant analysis (PLS-DA) model was performed to identify metabolites using SIMCA, version 17 (Umetrics). The PLS-DA models were subject to 100-fold permutations to evaluate performances and validated using the predictive ability of the model (Q2) and the ANOVA testing of cross-validated predictive residuals [84]. The most discriminating features were selected for building PLS-DA models. These models were again subjected to prediction accuracy and overfitting assessment by the corresponding tests (cross-validated predictive residuals) and comparison of the Q2 afterward. Features were selected based on their variable importance in the projection score. Univariate statistical analysis and heatmaps were performed using Metaboanalyst 5.0 [85]. The metabolite and lipid relative intensity differences between groups were tested using the *t* test or the Wilcoxon rank-sum test. The multiple testing corrections were controlled using FDR correction [86].

## Results

Recruitment began in May 2021 and concluded in May 2022. Of the 63 participants who were randomized, 6 dropped out before receiving the intervention. Consequently, 57 participants were analyzed based on the intention-to-treat principle.

The average age of the cohort was 42.39 (SD 12.83, range: 18‐65) years, and their average BMI was 26.27 (SD 4.26) kg/m^2^. Of the 57 participants, 86% (n=49) were females, and 79% (n=45) were New Zealand Europeans. There were more individuals with FC (n=39, 68.4%) than IBS-C (n=8, 31.6%).

Data analysis is complete, and full results have been submitted to a peer-reviewed journal, with an expected publication date of April of 2026. Meta-transcriptomics and PBMC analysis were not performed due to poor sample quality.

## Discussion

### Anticipated Findings

Several studies have demonstrated that the consumption of green kiwifruit improves laxation. However, there are few robust randomized controlled trials assessing the efficacy and mechanism of green kiwifruit ingestion for people with constipation. To our knowledge, this is the first study to evaluate the effectiveness of consuming 2 green kiwifruit daily for 4 weeks on abdominal pain as a primary end point and on colonic volume and chyme water content in individuals with constipation. In addition to physiome outcomes, the fecal microbiota composition and gene abundance, plasma and fecal metabolites, and plasma immune markers were also analyzed to understand the underlying mechanism.

Improvement in pain was chosen as a primary outcome to assess whether kiwifruit offers benefits beyond laxation. Specifically, the goal was to evaluate kiwifruit’s suitability as a natural dietary intervention for individuals who experience both pain and constipation. Several dietary fiber interventions, including wheat bran, psyllium, and inulin, are known to alleviate constipation but are not preferred by patients because they may exacerbate pain [20,87].

In addition, this study included a range of patient-reported outcomes to evaluate nongastrointestinal symptoms and mental well-being, aiming to determine whether kiwifruit positively impacts health beyond its nutritional composition. A recent study showed that habitual kiwifruit consumption improved the quality of life in people with constipation, supporting that kiwifruit provides benefits that extend beyond mere nutrition [21].

This study used an MRI protocol to measure colonic volume and water content after habitual kiwifruit consumption over 4 weeks. This approach replicates a more natural setting where kiwifruit is integrated into the regular diet of individuals with constipation. A previous MRI study used serial imaging to examine the effects of a high dose of kiwifruit in healthy adults, providing valuable insights into the potential mechanism [29]. Additionally, this study also takes into account the secondary outcomes of colonic volume and water content when calculating the sample size, aiming to optimize its clinical utility and reduce the chances of false positive results [88].

This study was an open-label design. Both participants and researchers were aware of the group assignments for kiwifruit and maltodextrin. Open-label studies are known to have potential biases in reporting and analysis, so several measures were implemented to minimize these risks [89]. Various questionnaires were used to reduce these risks by asking the same questions in different forms and styles. Additionally, only two researchers were informed about the group allocations, and multiple endpoints were assessed, including both subjective and objective outcome measures. Participants completed the subjective end points independently, without the presence of a researcher. Additionally, a blinded outcome analysis was conducted, and the trial’s protocol and statistical plan were finalized before this study commenced [90].

The inclusion criteria could be expanded to include individuals aged 65 years and older. This is a significant group worldwide [91,92]. Older adults experience changes in their gastrointestinal function, which alter taste, motility, hormone secretion, and anorexia [93], resulting in their exclusion from many studies. However, it is a growing population with a known increased prevalence of bowel irregularity. Future research could assess the effectiveness and the change in gastrointestinal function and fecal microbiota parameters following high-fiber food interventions. Such dietary interventions could be particularly beneficial for those experiencing bowel irregularities due to polypharmacy, defined as taking more than 5 medications [94].

### Conclusions

This study was the first to investigate the effectiveness of habitual consumption of kiwifruit (2 servings daily for 4 weeks) on abdominal pain, colonic volume, and colonic chyme water content. This study also assessed patient-reported outcomes and objective regional physiological, biochemical, and microbial changes that may aid digestion, absorption, and fermentation in order to understand the mechanism of action.

## Abbreviations

Atmo capsule: Atmo gas-sensing capsule
CONSORT: Consolidated Standards of Reporting Trials
EDTA: ehylenediaminetetraacetic acid
FC: functional constipation
FDR: false discovery rate
FFQ: Food Frequency Questionnaire
GSRS: Gastrointestinal Symptom Rating Scale
HPLC: high-pressure liquid chromatography
IBS-C: irritable bowel syndrome with constipation
LCMS: liquid chromatography-mass spectrometry
MRI: magnetic resonance imaging
MS: mass spectrometry
PBMC: peripheral blood mononuclear cell
PLS-DA: partial least squares projection-discriminant analysis
PROMIS: Patient-Reported Outcomes Measurement Information System
REDCap: Research Electronic Data Capture
ROI: region of interest
SF-12v2: Short-Form Health Survey Version 2 (12-item)
SPIRIT: Standard Protocol Items: Recommendations for Interventional Trials
TRUFI: True Fast Imaging With Steady-State Free Precession
WHO-5: World Health Organization - Five Question Well-Being Index
WMD: wireless motility device
